# DNA copy number changes in young gastric cancer patients with special reference to chromosome 19

**DOI:** 10.1038/sj.bjc.6600969

**Published:** 2003-06-10

**Authors:** A Varis, B van Rees, M Weterman, A Ristimäki, J Offerhaus, S Knuutila

**Affiliations:** 1 Departments of Pathology and Medical Genetics, Haartman Institute and Helsinki University Central Hospital, University of Helsinki, POB 400 (Haartmaninkatu 3, 4th floor), FIN-00029 HUS, Helsinki, Finland; 2 Department of Pathology, Academic Medical Center, Meibergdreef 9, 1105 AZ Amsterdam, The Netherlands

**Keywords:** gastric cancer, comparative genomic hybridisation, 19q, cyclin E

## Abstract

Only a few cytogenetic and genetic studies have been performed in gastric cancer patients in young age groups. In the present study we used the comparative genomic hybridisation (CGH) method to characterise frequent DNA copy number changes in 22 gastric cancer patients of 45 years or younger and three gastric cancer cell lines established from patients younger than 45 years. Analysis of DNA copy number changes revealed frequent DNA copy number increases at chromosomes 17q (52%), 19q (68%) and 20q (64%). To confirm the CGH results and to characterise the amplicon region on the most frequently amplified chromosome, chromosome 19, we carried out fluorescence *in situ* hybridisation (FISH) analysis and Southern blot analysis. Fluorescence *in situ* hybridisation with the bacterial artificial chromosome (BAC) clone mapped to 19q12 indicated a copy number increase in all eight tumour specimens studied. Southern blot analysis of six tumour specimens and three tumour cell lines, with five probes mapped to the 19q12–13.2 region, suggested cyclin E to be one of the candidate target genes in the 19q region for gastric cancer tumorigenesis. Cyclin E protein overexpression was verified in tumours with amplification on chromosome 19. Further studies are required to investigate the biological and clinical significance of 19q amplicon and cyclin E upregulation in gastric cancer of young patients.

Gastric cancer is the second most frequent cancer worldwide (Parkin *et al*, 1999), but in young age groups it is rather uncommon. Several studies have been published about gastric cancer in young patients aged 45 years or younger (Kokkola *et al*, 1996; Maehara *et al*, 1996; Theuer *et al*, 1996; 1998; Semba *et al*, 1998; Rugge *et al*, 1999; Haruma *et al*, 2000; Kath *et al*, 2000). These studies have suggested differences between clinicopathological and histological outcome in gastric cancer in young and elderly age groups. In young patients, gastric cancer has been suggested to arise with a more aggressive disease and poorer prognosis. Histologically the diffuse type of gastric cancer according to Laurén (1965) is suggested to be predominant in young age groups. *Helicobacter pylori* infection is assessed to be a risk factor also for young patients in both histological types.

Numerous cytogenetic and genetic aberrations have been described in gastric cancer in elderly patients. These include frequent DNA copy number gains in chromosomes 8q, 17q and 20q and losses in chromosome 4 by comparative genomic hybridisation (CGH) (Kokkola *et al*, 1997,^1998^; El-Rifai *et al*, 1998,^2001^). Several oncogenes, tumour suppressor genes and mismatch repair genes are assumed to be associated with the development and progression of gastric tumours. Two of the most investigated genes are *ERBB2* and *TOPO2A* making them possible prognostic markers of gastric cancer (Seregni *et al*, 2001).

Only a few cytogenetic and genetic investigations have been reported about gastric cancer in young patients (Maehara *et al*, 1996; Semba *et al*, 1998; Haruma *et al*, 2000; Rugge *et al*, 2000). So far no reports of sporadic/nonfamilial gastric cancer have indicated any specific genetic characteristic arising in young patients compared to elderly patients. Germline E-cadherin mutations have been found in patients with familial cases of the diffuse type gastric cancer (Gayther *et al*, 1998).

In order to find out whether the genetic changes in younger patients differ from those in older patients, we studied DNA copy number changes in gastric cancer of young patients. Using CGH, we analysed 22 tumour samples of patients aged 45 years or younger, and three gastric cancer cell lines established from patients who were younger than 45 years. To verify CGH results and to characterise the amplicon on the most frequently amplified chromosome, chromosome 19, we performed fluorescence *in situ* hybridisation (FISH) analysis for eight patients and Southern blotting analysis for six patients as well as for three cell lines derived from gastric carcinomas of young patients. In addition, we used immunohistochemical analysis for four patients.

## MATERIALS AND METHODS

### Cell lines

Three gastric cancer cell lines, TMK-1 (obtained from the Department of Pathology, Hiroshima University School of Medicine, Hiroshima, Japan), and MKN-7 and MKN-74 (obtained from the Second Department of Pathology, Fukushima Medical College, Fukushima, Japan) were used in this study (Table 1

**Table 1:** Clinical characteristics, CHG findings, Southern blotting and FISH results of tumour specimens and cell lines

**No.**	**Age/sex**	**Histology**	**Stage**	**CGH results/gains**	**CGH results/losses**	**Southern blotting results, amplified genes (ratio^a^)**	**Interphase FISH signals (BAC CTC-525D6)/average signals/nucleus)**
1	42/M	Intestinal	Advanced	5p, 8q, 17q12-qter, 19cen-q13.2	4, 5q, 6qcen-q22, 9p22-pter	—	—
2	39/M	Intestinal	Advanced	11qcen-q14, 19	4	—	2.5
3	41/M	Intestinal	Advanced	1q21-qter, 5p, 6cen-6p22, 6q16-qter, 7p, 8q, 11q13-pter, 13, 17/17q12–q21.32, 18/18p11.3, 20/20p11.22-pter/20q11.22-qter	1p31.2–p21.2, 2q21-qter, 4p, 4q24-qter, 5qcen-q31, 12q15-q22	—	—
4	44/F	Intestinal	Early	1q21-qter, 6p12.2-pter, 7q21.1-pter, 11qcen-q13, 12p, 16q11.2-pter, 17q22-pter/17qcen-q21.32, 19, 20	3, 4, 9p, 12q15–q22	—	—
5	44/M	Intestinal	Advanced	1q21-q23, 8p12-pter, 9p13-qter, 11q13, 17q, 18p11.3, 19, 20	18q	—	—
6	45/M	Intestinal	Advanced	19, 20	No losses	—	—
7	41/M	Intestinal	Advanced	1q21-q25, 3q26.2-qter, 6pcen-p22, 11q14-q12, 12, 17/17q, 19, 20q/20q, 22	4, 5q14.2-q23, 18q	—	—
8	42/M	Intestinal	Advanced	4p15.3-pter, 5p15.1-pter, Xq24-qter, 7, 8q, 10q, 11qcen-q14, 12p12-pter, 16, 17q, 19, 20q11.2-qter	4q, 6q25.2-p12.2, 13	—	2.7
9	39/M	Intestinal	Advanced	1q, 7, 8q, 10p, 17q21-qter, 18p, 19, 20	No losses	—	3.8
10	43/M	Intestinal	Advanced	1q, 2q34-qter, 7pter-q21,12q14-q15, 17, 18p, 19/19cen-q13.1, 20q12-qter	4, 5p13-q23.2, 9p24	*CCNE* (31)	>20
11	37/F	Intestinal	Advanced	1q, 5p15.1-pter, Xq/Xq22-qter, 8q, 9q, 13, 16p, 17cen-q23, 19, 20	No losses	*AKT2* (4.7)	3.0
12	45/M	Intestinal	Advanced	11, 12p11.2-p12, 19, 20q11.22-qter	No losses	No amplification	2.9
13	30/F	Intestinal	Advanced	Normal	Normal	—	—
14	28/M	Intestinal	Advanced	7p13-pter, 8q, 13q12-q14.11, 17q12–q21.31, 19cen-q13.2, 20q	No losses	—	5.5
15	39/M	Mixed	Advanced	1p31.2-pter, 1q, 2q34-qter, 5p13-pter, 7pter-q31.2/7p13-21, 8q12-qter, 9q21.12-qter, 11q12.2–q13, 12, 15, 17, 19, 20/20	11q22.3-qter	No amplification	2.5
16	42/F	Mixed	Advanced	16p	No losses	—	—
17	42/F	Diffuse	Advanced	Normal	Normal	—	—
18	39/F	Diffuse	Advanced	Normal	Normal	—	—
19	41/M	Diffuse	Advanced	Normal	Normal	—	—
20	38/M	Diffuse	Advanced	Normal	Normal	—	—
21	39/F	Diffuse	Advanced	8q, 13, 16p, 19p	8p, 9p, 16q	—	—
22	18/F	Diffuse	Advanced	Normal	Normal	—	—
23	43/M	Diffuse	Advanced	Sample not available	Sample not available	*CCNE* (3)	—
24	36/M	Intestinal	Advanced	Sample not available	Sample not available	No amplification	—
25	TMK-1 21/M		Poorly differentiated adenocarcinoma	1q21.2-qter, 5p, Xp21-qter, 10q13-pter/10q, 11q, 14, 19cen-q13.2, 20q	4,6q, 8q13-pter, 12p, 13, 18q21-qter, 21	*CEBPA* (4.1)	—
26	MKN-7 39/M		Well differentiated tubular adenocarcinoma	2p21-pter, 5p, X, 7p/7p15-pter, 8q22-qter78q24.1-qter, 9q32-qter, 11/11q13.5-q14, 14, 17, 18p, 19/19cen-q13.3, 20/20, 21q21-qter	1p32-q21.1, 4q24-pter, 6q15-q23, 8p21-pter, 9q21.32-pter, 10, 12q21.32-pter, 15q21-pter, 18q22-qter	*CCNE* (11) *CEBPA* (4.6) *AKT2* (2.9)	—
27	MKN-74 37/M		Well differentiated tubular adenocarcinoma	3q21-qter, 5, X/Xp, 6p12-pter, 7/7pcen-p15.1/7q22.2-q32.1, 11qcen-q21, 11q23.2-qter/11q24-qter, 12q21.2-pter, 13q32-qter, 14, 15q21-qter, 17, 19, 20, 21q22	1q21-p22, 1q42-qter, 4p14-qter, 6q15-qter, 8q11.21-q21.3, 9q34.11-pter, 18q21-q22.1	*BCL3* (3)	—

Straight line=sample not available or no indication for the Southern blotting of FISH.

a The threshold value for amplification=2.5.

). Cell lines were cultured under the recommended conditions. All three cell lines were established from male subjects, ages between 21 and 39 years. Two cell lines (MKN-7, MKN-74) were histologically classified as well-differentiated tubular adenocarcinoma and one cell line (TMK-1) as poorly differentiated adenocarcinoma.

### Patients

Archival paraffin-embedded specimens from 24 young gastric cancer patients were obtained from the Department of Pathology at the Helsinki University Central Hospital, Finland, the Department of Pathology at the Jorvi Hospital, Espoo, Finland, and the Department of Pathology at the Academic Medical Center, Amsterdam, Netherlands.

In all, 15 patients had intestinal type of gastric cancer, seven patients had diffuse type of gastric cancer, and two patients were diagnosed with mixed type of gastric cancer. The median age of patients was 39 years (range 18–45 years), and 14 of them were male patients. Two of the patients had family history (cases 11 and 24) of gastric cancer.

Gastric carcinomas were classified by an experienced pathologist. A distinction was made between the two main histological subtypes and in early and advanced carcinomas.

### Comparative genomic hybridisation (CGH)

For CGH, DNA was extracted from three cell lines, paraffin-embedded specimens from 22 young gastric cancer patients, and from the peripheral blood of a healthy male or female (reference DNA) using standard protocols. Comparative genomic hybridisation was performed and analysed as described previously (El-Rifai *et al*, 1997; Varis *et al*, 2001).

### Nuclei extraction and FISH

The nuclei from the paraffin-embedded tissues were extracted from the eight patients and two reference samples. Paraffin sections of 10*μ*m that were very rich in tumour cells were used in nuclei isolation of tumour samples. As a probe for the interphase FISH, we used bacterial artificial chromosome (BAC) clone CTC-525D6 (AC011474; Research Genetics, Huntsville, AL, USA). The nuclei extraction, FISH and analysis were performed as described previously (Hemmer *et al*, 2001), except origin of two reagents, biotin-14-dATP and Cot-1 DNA, which were purchased from Gibco BRL (Geithessburg, MD, USA).

### Southern blotting

Genomic DNA for Southern blotting was extracted from three cell lines and the sections of the frozen material of six patient samples using standard protocols. Southern blotting and hybridisations were performed by standard laboratory methods (Weterman *et al*, 1996). The percentage of tumour cells was estimated on an HE-stained section of the frozen material.

Southern blot analysis was performed using phosphorimager (Fuji) and AIDA software v.2.41. The signal intensity in the lanes containing tumour DNA was divided by the intensity obtained for the corresponding normal DNAs. Both signals were adjusted for loading differences and background using a *β*-globin control probe. The threshold value for amplification was 2.5. If corresponding DNA from normal tissue was not available (cell lines and case 23), intensity for normal DNA was based on the average intensity of signals from all other normal DNAs.

Five cDNA image clones for Southern blotting were obtained from the RZPD (Deutsches Ressourcenzentrum für Genomforschung GmbH, Heubnerweg 6, D-14059 Berlin, Germany). The clones were sequence verified and the corresponding inserts were isolated using appropriate restriction analysis and purification prior to labelling. The clones used were IMAGp998-O145473 (*CCNE*), IMAGp998B125762 (*CEBPA*), IMAGp998K066030 (*TGFB*), IMAGp998O131258 (*BCL3*) and IMAG p998B152412 (*AKT2*).

### Immunohistochemistry

Immunohistochemistry for cyclin E was performed using the monoclonal antibody CYE5 (Neomarkers, Fremont, CA, USA) in 1 : 40 dilution. Briefly, paraffin-embedded specimens were sectioned (4 *μ*m), deparaffinised, blocked for endogenous peroxidase activity by immersion in 0.3% H_2_O_2_ in methanol for 20 min and heat treated at 100°C in (pH 9) for 10 min. Nonspecific binding sites were blocked in 5% normal goat serum for 10 min following the incubation for 1 h with the primary antibody at room temperature. The Powervision+poly-HRP detection system (ImmunoVision Technologies, Co, Daly City, CA, USA) was used to visualise the antibody binding sites with 3,3-diamino-benzidine+ as a chromogen. Sections were counterstained with haematoxylin.

## RESULTS

### Comparative genomic hybridisation

**Figure 1:**
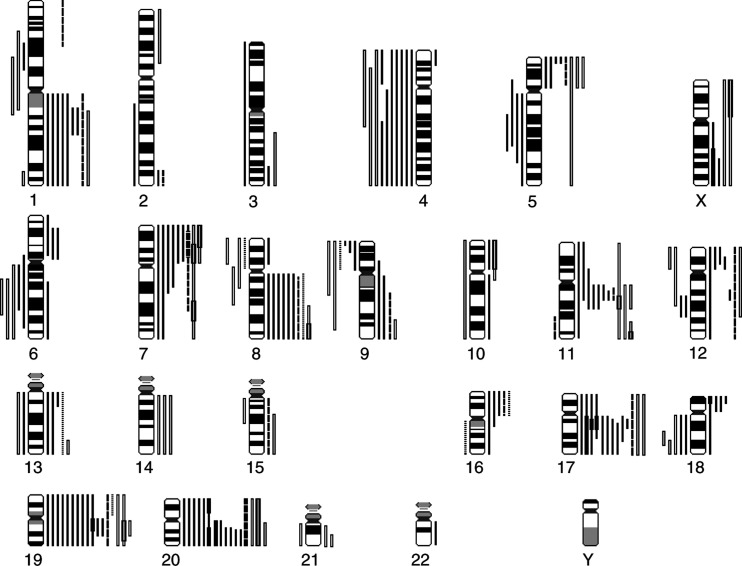
Summary of gains and losses in 22 tumours of young gastric cancer patients and three cell lines. Gains are shown on the right sides of chromosomes and losses on the left sides. Intestinal tumours, solid lines; mixed tumours, broken lines; diffuse tumours, dotted lines; cell lines, open bars. High-level amplifications are marked with a thick bar.

To identify DNA sequence copy number changes in young patients with gastric cancer, 25 gastric carcinoma patient samples and three cell lines were included in CGH analysis. A summary of the gains and losses detected by CGH is shown in Figure 1

. Detailed clinical and karyotypic data are presented in Table 1, which is available as a supplement on the BJC web site, www.nature.com/bjc/. DNA copy number changes were more frequent in intestinal cases (13 out of 14 cases, 93%) than in diffuse cases, of which only one of the six cases was abnormal (17%). DNA copy number changes were detected in both mixed gastric carcinoma and all three gastric carcinoma cell lines. The most common changes were gains at chromosome 19q (17 out of 25, 68%), at chromosome 20q (16 out of 25, 64%) and at chromosome 17q (13 out of 25, 52%). Losses were frequent at chromosome 4 (10 out of 25, 40%).

In the group of intestinal carcinomas DNA copy number increase was frequently observed in chromosomal arms 20q (86%), 17q (71%) and 19q (71%). Frequent losses were seen at 4q (50%) and 5q (29%). High-level amplifications were present on the q-arm of chromosome 17, p-arm of chromosome 18, q-arm of chromosome 19 and in chromosome 20. In all three cell lines derived from young patients gains were detected for chromosomes 5, 11, 19 and 20. High-level amplifications were again seen on chromosomes 19 and 20, but also in chromosomes 7 and 11.

### Interphase FISH

In order to confirm the results obtained by CGH, eight gastric cancer cases of the intestinal type (cases 2, 8, 9, 10, 11, 12, 14 and 15) with amplification on chromosome 19 or the 19q arm and two normal stomach tissues were investigated by FISH using BAC CTC-525D6, which maps to 19q12, as a probe. This also allows for specification of copy number changes in the tumours examined. On average two signals were observed in two normal stomach tissue preparations. One case (case 10), which showed high-level amplification on chromosome 19 by CGH, had an average of over 20 copies per nucleus for BAC CTC-525D6. Other samples of tumor tissue also showed increases in copy numbers for BAC CTC-525D6 the range being between 2.5 and 5.5 copies per nucleus (Table 1).

### Southern blotting

To characterise the amplified region on chromosome 19 in more detail, Southern blot analysis was performed for six gastric cancer tumours (cases 10, 11, 12, 15, 23 and 24) of which frozen material was available and five normal controls (cases 10, 11, 12, 15 and 24) of the same patients. In addition, three gastric cancer cell lines (MKN-7, MKN-74 and TMK-1) derived from young patients were analysed. Five cDNA clones that mapped to the 19q12–13.2 region [*CCNE*, *CEBPA*, *AKT2*, *TGFB* and *BCL3*, mentioned in the order of their location on chromosome 19 (Table 1)] were used as probes. The highest amplification level was seen for *CCNE* (cyclin E) in case no 10 (ratio 31) and cell line MKN-7 (ratio 11). Amplifications were also detected at a lower level for *CEBPA* and *AKT2*. Amplification of *BCL3* was only detected in one case and *TFGFB* was not amplified in any of the samples.

### Cyclin E expression

**Figure 2:**
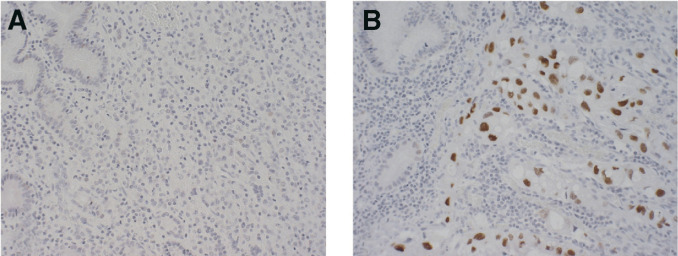
Immunohistochemical analysis of cyclin E protein expression in gastric cancer tumours. (**A**) Diffuse type of gastric cancer tumour without 19q amplicon, no detection of cyclin E overexpression and (**B**) Intestinal type of gastric cancer with 19q amplicon, increased nuclear staining of cyclin E detected.

**Figure 3:**
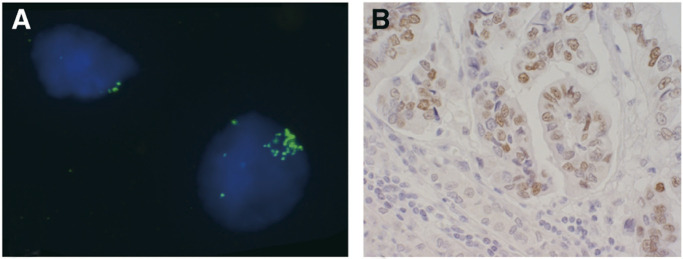
Fluorescence *in situ* hybridisation and immunohistochemical analysis of intestinal type of gastric cancer tumour (Case No. 10). (**A**) Fluorescence *in situ* hybridisation image of hybridisation with BAC probe targeting the 19q12 region (green signals). Normal nucleus with two signals on the left side and nucleus with amplification on the right side. (**B**) The same tumour indicating positive staining of cyclin E protein.

Four samples from young gastric cancer patients were immunostained for cyclin E. Two of the cases (cases 17 and 19, Table 1) without any 19q abnormalities did not show overexpression of cyclin E (Figure 3

), whereas the other two cases (cases 10 and 14, Table 1) with a 19q amplicon detected by other methods showed positive staining for cyclin E (Figures 2

and 3).

## DISCUSSION

In the present study we used CGH to characterise DNA copy number changes in young patients with gastric cancer. The most frequent cytogenetic aberrations were gains seen at 17q (52%), 19q (68%) and 20q (64%). DNA copy number changes were mostly detected in intestinal or mixed types of tumour, which is in agreement with our previous studies of gastric cancer tumours (Kokkola *et al*, 1997,^1998^). Our study of intestinal type of tumours, in which the mean age of the patients was 67 years, showed frequent gains in chromosomes 8q (45%), 17q (41%) and 20q (55%) and losses in chromosomes 4q (32%) and 18q (41%), whereas no abnormalities were observed for chromosome 19. Our results of DNA copy number changes in older age group accord well with the results of the other CGH studies in older gastric cancer patients (Van Dekken *et al*, 2001; Wu *et al*, 2001). However, some dissimilarities between the published gastric cancer studies are found, for example, frequency of abnormalities in chromosome 19. In the study of Van Dekken *et al* (2001) gain at 19q was involved in 30% of cases in older age group. Still, the frequency of the 19q gain observed in our present study on young patients is significantly higher than previously indicated in gastric cancer.

The most remarkable difference we detected in relatively young patients compared to previous investigations was the frequency of the 19q amplicon. Frequent DNA sequence copy number increases at chromosome 19 in other cancers have been described in myeloma and plasmacytoma, small cell lung cancer, adrenocortical tumours (childhood) and pancreatic endocrine tumours (Knuutila *et al*, 1998, updated 2002, http://www.helsinki.fi/emg).

To confirm and narrow the target region on chromosome 19q, we performed FISH and Southern blotting analysis. The FISH study using a BAC probe which, mapped to 19q12, indicated a copy number increase in all studied tumour specimens in this region. One of the most likely candidate genes in this region is *CCNE* (cyclin E). Amplification of the cyclin E gene was identified in one cell line and two tumour specimens by Southern blotting. Three other genes, *CEBPA*, *BCL3* and *AKT2*, showed lower amplification status than cyclin E, and Southern blot did not show any amplification in the *TGFB* gene. These facts indicate that the critical target segment is more probably located in the region between 19q12 and the centromere than in the 19q13.1–13.2 region. Immunohistochemical analysis of cyclin E protein demonstrated overexpression of cyclin E in tumours with 19q amplicon, but not in tumours without 19q abnormalities. Further studies are needed to investigate whether this region includes other genes, in addition to cyclin E, critical for gastric cancer development and progression. On the other hand, coamplification of neighbouring genes in the target segment, without upregulation, may have occurred (Varis *et al*, 2002). Previously a few reports have been published about findings of cyclin E upregulation in gastric cancer of older patients (Akama *et al*, 1995; Sakaguchi *et al*, 1997; Lin *et al*, 2000).

Overexpresssion of cyclin E has been suggested to correlate with the *P53* expression, progression of gastric carcinoma and poorer prognosis (Sakaguchi *et al*, 1997). Cyclin E has an important role as a cell cycle regulator. After forming a complex with cdk2 cyclin E regulates the transition from the G1 phase to the S phase in the cell cycle and, as such, may play a role in gastric carcinogenesis. In addition to gastric cancer, amplification and overexpression of cyclin E have been detected in several other carcinomas, including oesophageal cancer, ovarian cancer and sarcomas (Akama *et al*, 1995; Donnellan and Chetty, 1999; Lin *et al*, 2000).

In this study slight discrepancies between used methods may exist, caused by the fact that the probes used in FISH and Southern blot analysis are not overlapping and thus cover another genomic region, although all map to the 19q12–13.2 region. The BAC clone CTC-525D6 is mapped to 19q12, but it does not cover the region of *CCNE*. In addition, CGH and FISH were performed on the same paraffin-embedded material with enriched tumour cells, whereas frozen material with varying proportions of tumour cells was used for Southern blot analysis. This fact may mask the presence of amplifications in Southern blot analysis. Nevertheless, it is important to underline that high-level amplifications at 19q were observed by all methods used in this study.

Most of the patients had advanced stage of the tumour and for this reason we were not able to compare the correlation of chromosomal aberrations with the stages of the tumours.

The target genes in the other frequently amplified region, 17q12–q21, are well studied. In our previous research in gastric cancer xenografts and cell lines, amplified and overexpressed genes detected in this region were the well-known *TOPO2A* and *ERBB2*, and one uncharacterised EST (Varis *et al*, 2002). *TOPO2A* and *ERBB2* are considered to be possible target genes in cancer therapy, especially in breast cancer (Cuello *et al*, 2001; Harris *et al*, 2001).

Amplification of 20q has been reported in different kinds of tumours. Several amplified and overexpressed genes have been identified on this chromosome, such as *BTAK* in gastric, breast and ovarian cancer, *MYBL2* and *ZNF217* in breast and ovarian cancer, *NABC1* in breast cancer, *TGIF2* and *PTPN1* in ovarian and breast cancer, and *AIB1* in gastric, ovarian, pancreatic and breast cancer (Collins *et al*, 1998; Knuutila *et al*, 1998; Ghadimi *et al*, 1999; Forozan *et al*, 2000; Imoto *et al*, 2000; Sakakura *et al*, 2000, 2001; Tanner *et al*, 2000). In addition to *BTAK* and *AIB1*, some of the other known oncogenes in the 20q region may also be target genes in gastric cancer.

To conclude, our study revealed frequent DNA copy number changes in young patients with intestinal and mixed types of gastric cancer and in gastric cancer cell lines established from young patients. The most frequently observed abnormalities were gains and high-level amplifications on chromosomes 17q, 19q and 20q. In addition, we identified one amplified and overexpressed target gene, cyclin E, in the most frequently amplified target segment of chromosome 19. Further studies would elucidate the biological and prognostic role of 19q amplicon and cyclin E upregulation in young patients with gastric cancer.
